# Obstetrics, and Diseases of Women and Children

**Published:** 1860-05

**Authors:** Wm. B. Atkinson

**Affiliations:** Lecturer on Obstetrics, and Diseases of Women and Children


### OBSTETRICS, AND DISEASES OF WOMEN AND CHILDREN.

BY WM. B. ATKINSON, M.D.,

LECTURER ON OBSTETRICS, AND DISEASES OF WOMEN AND CHILDREN.

### I.—*Post-Partum Hemorrhage: Its Treatment.* By T. G. THOMAS, M.D.

Having given in our last number an abstract of a lecture by Dr. Thomas, on the *Prevention of Post-Partum Hemorrhage*, it is eminently proper that its treatment should follow next in order.

The varieties of hemorrhage, as occurring in the lying-in chamber, are: internal, external, immediate, remote.

The sources of hemorrhage are from ruptured vessels of the vulva, from the os and cervix uteri, from the uterus and placenta.

The causes are inertia uteri; uterine exhaustion; sudden evacuation of the contents of the womb; mental emotion; excited circulation; adherent placenta; distention of the uterus by blood, or another child.

The indications to be fulfilled are, to prevent death by syncope or exhaustion; remove anything preventing uterine contraction; force the uterine fibres to contract. To fulfill the first indication, we must admit a current of air; lower the patient's head; give brandy and ammonia, if necessary; let an assistant stand ready to dash cold water into the face. To accomplish the second indication, pass the hand into the uterus up to its fundus, and by gentle titillation of the uterine fibres, cause them to contract, and remove everything which will be liable to prevent a full and permanent closure of the uterine vessels. For the third indication, keeping one hand in utero, grasp and knead the fundus with the other; have a stream of water poured on the abdominal walls, from a height of about four feet; give full doses of ergot; pass lumps of ice into the vagina, and, if necessary, into the uterus, and also throw enemata of ice water into the rectum. To cause the contraction to be permanent, do not allow the binder to be applied for some hours, but keep the uterus constantly in the grasp of a trusty assistant, for twelve hours or longer if necessary; apply the child to the breast; keep up the action of ergot; should vomiting exist, give it per rectum; rouse the dormant energies of the uterus by an enema of spirits of turpentine with mucilage or oil; let the patient drink cold and acid draughts. Do not annoy the stomach, and lose this important avenue to the system by the use of gallic acid, lead, or opium, *which do not fulfill the indication*. The time for these may come, but this is not it.

The after-treatment will be indicated by the symptoms: as supplying artificially the brain and other important organs with blood; preventing death from exhaustion; quieting the perturbed state of the nervous system. The first will be fulfilled by keeping the head very low; raising the legs and arms in the air, supporting them thus by pillows, etc.; applying tourniquets to the femoral and brachial arteries, and thus cutting off for a time the supply to the extremities; gentle pressure by roller bandages applied the whole length of the arms and legs; and finally, transfusion. The second, by the administration of brandy, ether, or ammonia; after the most pressing symptoms have passed, by beef essence, milkpunch, or eggs beaten up with wine; quiet, solitude, and darkness. The third, by opium in no stinted dose, four grains of the powder, or a drachm of the tincture, every half hour or oftener, watching the effect, not the amount; morphia applied by the hypodermic method. The sharp point of the syringe of Dr. A. Wood, of Scotland, is passed under the skin, giving very little pain, and ten drops of the solution of sulphate of morphia are injected. Narcotic symptoms show themselves in two or three minutes.—(*N. Y. Jour. of Med.*, March, 1860.)

II.—*Special Position and the Obstetric Binder as Aids in the Treatment of Impeded Parturition.* By ROBT. HARDEY.

The position advocated is the sedentary on chairs, first proposed by the late R. M. Craven, Esq., of Hull, in 1827, and which, from that time, has been adopted by Mr. Hardey in all cases where the difficulties to be overcome demanded more than ordinary efforts for delivery. All agents which sustain and increase motor force are real benefits to the parturient female. Two of the most valuable are the sedentary posture and the obstetric binder. The plan adopted is to secure the fronts of two chairs to each other, and separate their backs, from one and a half to two feet; to place the patient well over or between these, with her knees firmly pressed against the side of the bed, her chest fixed by holding on to the foot post of the bed, and her feet placed firmly on the floor. The accoucheur sits or kneels behind the patient, who remains on the chairs till the difficulties are overcome, evidence of which is obtained by the emerging of the parietal bones from behind the perineum. The woman is then removed to bed, and finally delivered in the ordinary position. Before seating the patient, her abdomen is to be carefully sustained by a broad binder. In every case, before adopting the sedentary posture, the part presenting should be somewhat within the pelvis, and the os uteri half dilated. The practice is contra-indicated by impending exhaustion, inflammation, convulsions, hemorrhage, version presentations, and extreme pelvic obliquity. The author claims that he thus secures the aid of gravitation; the highest amount of motor energy; the bringing of the abdominal and pelvic axes into the same obstetric plane; the imparting of great support to the fundus uteri in its contractions, by the binder. He recommends this plan for its simplicity; freedom from danger; great potency; its testing the ability of nature to accomplish the delivery at a period sufficiently early to enable the accoucheur to decide on the use of instruments before material damage has been sustained by the maternal tissues; and its being a great economist of professional time.

It was, however, not intended to supersede the position on the left side, which is admirably suited for the greater number of ordinary labors.—(*Obstetrical Society; Med. Times and Gazette*, March 17, 1860.)

III.—*Influence of the Mother's Mind on the Fœtus in Utero.* By JOHN M. WATSON.

After a very interesting investigation of this matter, Dr. Watson sums up thus:—

Instinct may produce changes in animal organisms in a variety of ways.

The soul or mind acts through a material organization, which may be changed by its influence. The human organism undergoes very striking changes when the mind is kept in abeyance by animal passions and bestial habits. The passions may produce lesions of structure, various diseases, and often death. The mind when improperly employed may develop premature puberty, premature ova, and premature menstruation, to which may succeed premature conception; and all the energies of both soul and body being directed to this uterine function, may not certain images, under strong mental excitement, be transferred to the embryo? The embryo is sometimes altered or changed in organization in various ways, through the mother's mind or imagination.—(*Nashville Jour. of Med. and Surg.*, Feb. 1860.)

IV.—*The Uterus and its Inflexions.* By PROFESSOR ROKITANSKY.

1. The vaginal portions of the uterus of a multipara, and the connected vaginal roof, is formed out of a duplicature or folding-in of the vagina, in which the lower end of the uterus takes a part. So soon as the uterus has passed into the vagina, the latter surrounds it like a ring, forming an intussusception. In front, the doubling is shorter, and is attached by loose cellular tissue. Hence, the anterior lip is thicker, and the vaginal roof more shallow. After many labors the distinctions become less. On section of the uteri of young persons, it is seen that the vagina, after it has formed the duplicature constituting the roof, is continued into the uterus. The mucous membrane and the layer of the two grow into the corresponding tissues. A second, outer, loose, muscular, longitudinal, fibrous layer of the vagina goes outside, over the duplicature, and spreads over the body of the uterus. In more mature uteri, and those which have been pregnant, there is interposed between the mucous membrane of the cervix and the longitudinal, muscular layer, a richer mass of uterus which ends in a point in the anterior lip of the vaginal portion. At the anterior side of the uterus runs the round ligament, separating into two muscular bands; the upper run together at the fundus, the lower under an angle in the neighborhood of the os uteri internum, thus forming a lozenge-shaped space. At the seat of union of the lower bands, in uteri of this description, there strikes a band of about an inch broad, in the form of a bow, the fasciculi of which fix and enlarge the duplicatures. On the posterior wall of the uterus, the ascending band is continued over the cervix into the vagina; or there proceed, also, from the end of this band, in the neighborhood of the os uteri internum, two strips of the form of a sharp bow, to the vagina. The strong mucous membrane of the cervix, and the thicker connective tissue on the posterior wall, do not terminate at the os internum, but, becoming thinner, go on to the body of the uterus. This forms the support of the mass of the uterus, and the foundation of its upright position, and shares essentially in flexions. Inflexion of the uterus, forward or backward, always falls in the region of the os internum. Flexions of the cervix seldom happen. The stratum of connective tissue is always found less thick, looser, thinner, and even wasted away. Hence, anteflexion is more frequent, and in less degree, as it grows to infraction; retroflexion is less frequent, but oftener in extreme degree, and very seldom grows to infraction. Anteflexion, moreover, most commonly appears in the virgin uterus, or at least it is apparently in no relation with labor; retroflexion, on the contrary, hardly ever arises but after repeated labors, or abortions.—(*Allg. Wiener Med. Zeitung*, No. 17, 1859; *Brit. and For. Med.-Chir. Rev.*, January, 1860.)

2. *On the Origin of Uterine Flexions.* By PROF. VIRCHOW. This writer sums up thus: At the seat of infraction there is found no primary alteration of tissue. Simple relations of pressure, distinguished from actual tumors, cause no anteflexions, but mostly retroflexions. Filling of the bladder and rectum cause distinct changes of position of the uterus. The changes are no longer possible when the body of the uterus is fixed at a certain height. In original shortening of a lateral ligament, there are found in children only lateral dislocation and inflexion; in persons beyond puberty, anteflexions. Anteflexions are more frequent in normal, retroflexions in pathological conditions of the uterine walls.

Hence, he draws the following therapeutical deductions: In the history of flexions there is a period of simple predisposition, one of simple flexion, and one of flexion complicated with various inflammatory processes; the predisposition is frequently given by partial forms of peritonitis, which appear with colicky attacks, and are apparently very difficult to mitigate; long retention of urine and fæces favor the formation of flexions, especially at the menstrual period, that of child-bearing, etc.; enlargements of the uterus, especially when united with relaxation, quickly cause flexions, and the removal of these may materially alleviate them; hence, the antiphlogistic treatment of uterine catarrh, and most careful watching of the menstrual and puerperal processes are necessary; a complete removal of anteflexion seems in the highest degree doubtful, while in retroflexion it may be expected. When flexion is connected with consecutive processes, as endo- and peri-metritis, a careful local treatment is necessary.

Virchow remarks upon the views of Rokitansky, as given in the preceding article, and does not regard atrophy at the seat of infraction as primary or essential, for in anteflexions of infantile and maiden subjects there is not the slightest alteration of the uterine wall to be found. He does not consider the anatomical description of Rokitansky as in all points correct; thus, the mucous membrane of the cervical canal cannot be called callous, for it is here relatively thin; it more resembles granulation tissue. The fibro-muscular tissue of the uterus is found as well in the body as in the neck; it contains more muscular fibres and vessels in the body, and more fibrous connective tissue in the neck. Toward the mucous membrane the muscular fibres in both places cease, and there is found a distinct, apparently thick, but in the normal state by no means thick, submucous layer.—(*Ibid.*)

V.—*On the Difficulty of Diagnosis between Pregnancy and Tumors of the Abdomen.* By ROBT. C. CROFT, Esq., L.R.C.P., Edin., etc.

There are few cases likely to prove more embarrassing, especially to the young practitioner, than those in which he is called to determine the nature of abdominal enlargements. These depend upon so many causes, and are often so masked, that it is frequently impossible to give an opinion with safety. When such protuberances exist in the male, the diagnosis, as far as pregnancy is concerned, is perfectly clear, but in the female, whenever her abdomen begins to enlarge, and if she be at the child-bearing age, pregnancy is at once suspected as the cause. There are two classes of women who come under our care who frequently baffle the medical man in his examination. These are unmarried women, who have hence no legal right to become mothers; and those who, being married, are eagerly desirous of becoming mothers. The first class will strongly deny symptoms that may exist, and place every obstacle in the way of the obstetrician; the second, by her hope of being pregnant, will be induced to describe imaginary symptoms. To illustrate these remarks he gives the following case:—

Mrs. ——, aged forty-two, married nineteen years. No children or miscarriages; in bad health; has had fistulæ in ano. Six years ago was struck in the right lumbar region by the pole of an omnibus, from which she has suffered more or less ever since. Two years ago, injured again in the right groin. When requested to see her she believed herself *enceinte*, and was suffering from distressing sickness. Felt, six months previous to the visit of her physician, a peculiar sensation in the region of the uterus. The catamenia appeared regularly till two months ago, when they ceased entirely; the breasts were full and painful, and there was a tumor in the lower part of the abdomen.

The next visit she was in bed; her countenance pale and anxious, breasts full and tense, tender to the touch, containing milk, which could be extracted; nipples large and sore; areolæ raised and darkened; abdomen tense and tympanitic, and just above the symphysis pubis was felt a hard substance, the size of an orange, in the pelvis, and well defined, nearly in the middle line of the abdomen, and inclined to the *left* side; no discharge from the vagina. By a stethoscopic examination a peculiar whirring sound was heard, like the placental murmur, to the *right* of the middle line above the pubis. From this time for three weeks no pain save in the breasts and nipples; the sickness constant; the only relief obtained was from ice. After a time the sickness abated, and food could be taken. At her next menstrual period she suffered, for three days, violent uterine pains; no discharge, the tumor seemed to have increased in size, and to be higher in the abdomen.

She was seen by other practitioners, who seemed equally doubtful as to the existence of pregnancy. But slight changes were observed for some five weeks. Kiesteine, on every examination of the urine, was detected. Now a new symptom arose; profuse spontaneous salivation came on. An examination, per vaginam, showed the cervix uteri shortened, the os opened, the lips puffy and swollen; on pressing upward with the finger, behind the symphysis, a hard substance was felt, very much resembling the head of a child at a late period of gestation. Dr. Robert Lee and Mr. Havers saw her, and while admitting the presence of most of the symptoms of pregnancy, yet declined to give a decided opinion. The only certain sign, the pulsation of the fœtal heart, could not be detected.

December 17th.—The condition of the patient pretty much as before; the tumor appearing to occupy the whole of the upper portion of the right half of the abdomen as high as the ribs, and extending deeply into the lumbar region. On pressure, a substance, like the gravid womb, was felt, and *in it* a distinct movement as of a fœtus. With the stethoscope, the peculiar sound was heard as before, with a cooing as of a dove. Upon shifting the instrument a little higher up, the fœtal heart was heard distinctly. Being now certain of the existence of pregnancy, the tumor was attacked, by directing the nurse to rub it gently, night and morning, for a quarter of an hour, with liniment of iodide of potassium. By the first of the succeeding March, the patient was still vomiting freely; the abdomen large, tense, dull on percussion; the umbilicus prominent; the ensiform cartilage pressed out; the abdomen measuring forty-two inches round. When a contraction of the uterus occurred, the tumor was very apparent.

May 10th.—After a severe labor, delivery took place. The placenta having been removed, a large firm mass was felt, seeming to occupy nearly the whole of the abdomen, and extending crosswise from the left ilium to the ribs on the opposite side. By firm pressure, the greater portion of the tumor sunk into the pelvis, leaving a large, round, hard ball loose in the abdomen. It was evident that the womb had been distended by the effusion into it of a quantity of blood. From this time all went well, the tumor remaining about the size of a large cricket ball, seemingly attached by a very small pedicle.—(*Lancet*, January 28, 1860.)

VI.—*Practical Remarks on Fœtal Auscultation.* By R. DRUITT, L.R.C.P., London.

The readers of the *Medical Times* have been startled by the announcement, on the part of one of the most learned physicians in the world, that the fœtal heart cannot be heard before birth; that if certain sounds be heard, said to be those of the fœtal heart, there is no certainty attainable that they are what they are supposed to be; and that the commonly described sounds are illusions, and exist only in the minds of the listeners. First of all, as to the facts asserted, and the reasonableness of them. To many physicians it is absolutely certain, that at most times, in women pregnant of a live child, especially after the fifth month, there can be heard, over some part of the enlarged uterus, a small and distant, but often remarkably distinct heart-beat, varying from 140 to 180 in the minute. By a heart-beat is meant a double beat, of one louder and more pronounced, and another shorter, immediately succeeding, and less pronounced.

It is said to be incredible that the sounds of the fœtal heart can reach the ear through so great a mass, consisting of uterine tissues, vessels, and the limbs of the child. But the thickness of the uterus and abdominal walls is not great, and not to be compared in sound-deadening qualities to a common pillow. But who doubts that the ticking of a watch can be heard under a pillow? The writer has heard his own watch through the thickness of sixteen folds of blanket. There is, also, no real difficulty concerning the counting of so large a number of pulsations as 160 in a minute. The breathing of dying children may be distinguished at 180; the pulse at 240. The ticks of a lever watch are five to a second, and may most easily be counted at 240 per minute. When the piano is played rapidly, the ear can readily recognize 720 sounds in a minute.

There is no doubt that a woman may be pregnant of a living child, and yet that at times the fœtal heart-sounds not be discovered even by one accustomed to the search. Hence, the absence of the sounds can never be taken as a proof that the fœtus is dead, or that pregnancy does not exist.

Again, sounds may be feebly heard, so that the observer cannot say that they are not heart-sounds; or other sounds may be mistaken for those of the fœtal heart. If the sounds are not confined to a small circle, or are synchronous with those of the maternal heart, they should not be suspected to be fœtal.

Of all the signs which distinguish the enlarged uterus from other tumors, none are more valuable than the following: the uterus, like other hollow viscera, has a regular peristaltic motion, continuous throughout pregnancy, (and after delivery,) and consisting in periodic contractions, which cause a moderate but decided tension of the organ, and are followed by flaccidity and repose. No other tumor, not tympanitic, can do this; and, during the fits of contraction, the shape, dimensions, and outline of the organ are unmistakable. When about to auscultate, gently shampoo or roll the abdominal parietes over the womb, till it becomes hard and resisting. This is the moment for auscultation. Put the stethoscope on the womb, and perpendicular to its surface. Search carefully on the horizontal line on a level with the anterior-superior spine of the ilium; beginning on the left side, then a little above and below; if unsuccessful, go to the right side. Take care that the attitude is easy, and produces no rushing sound in the ears.—(*Medical Times and Gazette*, January 21, 1860.)

VII.—*The Mental Peculiarities and Disorders of Childhood.* By Charles West, M.D., etc.

No one can have watched the sick-bed of a child without being struck by the almost unvarying patience with which its illness is borne, and the extremity of the peril from which, apparently in consequence of that patience, a complete recovery takes place.

No sorrow, gloomy foreboding, remorse, disappointment, nor anxiety, depresses the spirits and weakens the vital powers. Death, in general, brings no alarm. To keep the child happy, remove all causes of alarm, suffering, and discomfort; modify the treatment so as to escape a struggle with waywardness, and if death appears imminent, look at it from a child's point of view; all these are duties of the utmost importance both for physician and parents.

The mind of the child is feebler than that of an adult, but is proportionally active, and vivid in its imaginations. The child who dreads solitude, and asserts that it hears sounds or sees objects, often tells a literal truth. The sounds have been heard; in the stillness of the nursery, the little one has listened to what seemed to be a voice calling it; or, in the dark, phantoms have risen before its eyes, and the agony of terror betrays an impression far too real to be explained away, or to be suitably met by hard words or unkind treatment.

Disorder of the cerebral functions greatly exaggerates these impressions. Hence, the circumstances surrounding a child, whether in health or disease, are of vast importance, and should never be lost sight of in the treatment.

The passions, too, of a child, are exaggerated as a general rule. Reason as yet does not govern its caprices. There is also an intense craving for sympathy, which sometimes becomes quite uncontrollable, and requires as much care and judicious management as in the case of an adult monomaniac. As in diseases of the body, so in affections of the mind in early life, the power of repair causes a constant hope, which is not to be indulged in the cases of adults. Dullness, apathy, and cerebral disturbance, have, therefore, not so grave an import as at a more advanced age. The whole of the intellectual energy is expended on the child's commerce with the world; his relations to it are disturbed, hence, the want of interest, the slowness to resume the lively prattle after sickness should not be viewed with too much apprehension. The memory of a child is feeble, and when recovery takes place, it has to learn again its old lessons, and with weakened faculties. This process will extend over a longer time in proportion as it was younger at the time of the illness.

One thing should be remembered, in protracted illness, even when unaccompanied with disorder of the brain—the sense of hearing may be impaired, and this may be one cause of the child's dullness. The arrest of development, or the positive retrocession of the mental faculties, is of far less import than any perversion of the moral powers. The child who, in spite of dullness, manifests the ordinary childish feelings, may be much improved by judicious training. We must also, in forming an estimate of these capabilities, consider the accompaniments of the sickness. Convulsions or serious cerebral disturbance will correspondingly impair more profoundly the intellectual powers, and retard the recovery. It must be remembered that a very large number of children whose progress has been arrested at an early age are allowed to grow up without any culture, and much of their dullness may be due to neglect. Apart from congenital instances, where mind and body are alike arrested in development, or are alike feeble and deformed, the state of the moral powers are more important as a guide to the prognosis than the condition of the intellectual. Want of affection, mischief, spite, causeless rage, are less hopeful than intellectual dullness, and the first step should be the establishment of moral control. It should be borne in mind that the heart may break or reason fail under causes seemingly insufficient, and the griefs of childhood may be as overwhelming as those of the strong man. The intellectual powers should never be overtasked. Thus may be laid the foundation of hydrocephalus, or the tubercular cachexia, the destruction of the nervous system or serious injury to the moral character.

Occasionally, children exaggerate their ailments, or feign those which have no existence, and they will put up with scanty fare and painful treatment as long as they can engross attention, and be the centre around which the household turns.—(*Med. Times and Gazette*, Feb. 11, 1860.)

### VIII.—*On the Employment of Iodide of Potassium in Diseases of the Brain in Children.* By JOHN COLDSTREAM, M.D., F.R.C.P., Edin.

It is now upwards of twenty years since iodide of potassium was commended by Rœser and others, as a remedy of special power in hydrocephalus. It is surprising how few seem to recognize its value, and what slight references are made to its employment in the various works on the diseases of children. In all cases when, from the course of the symptoms, there is reason to believe that the central organs of the nervous system, or their envelopes, are in any degree affected with strumous inflammation, (tubercular cerebritis, or meningitis,) or its consequences, after moderate purgation, the writer is in the habit of employing the iodide of potassium in doses of from half a grain to three grains, every three or four hours, in some carminative water, and continuing it for many days, according to the symptoms, or until convalescence is fully established; and with the occasional use of blisters to the shaven scalp, he believes he has produced more prompt and decided effect upon the disease than by any other treatment. When the opportunity has been afforded of commencing the use of this remedy early, it has appeared to arrest the progress of the disease *rapidly*, so that the effects of effusion, indicated by squinting and convulsions, have not supervened. In less favorable circumstances, where considerable prostration had succeeded great febrile action, where starting and squinting had become prominent symptoms, in not a few instances, the free use of the iodide of potassium has been followed by amendment and complete recovery. In such cases it should be given in larger doses, even to four grains several times a day, to children of from four to eight years of age.

The medicine is very seldom refused by the patient, nor does it increase the nausea so frequently existing in the earlier stages of the disease; nor has it induced salivation, which seems sometimes to follow its use in other ailments. Although it is more especially useful where there exists more or less of the scrofulous diathesis, yet it has been found of service in cases where no taint was present.

The writer is not prepared to assert that this agent is more useful than calomel in *all* cases of inflammation of the brain and its appendages. When we have to treat robust and full-blooded children, in whom there is reason to believe that the threatened disease of the nervous system stands more or less directly connected with preceding disorder of the digestive organs, there is no doubt of the superior efficacy of the mercurial treatment, combined with antimonials and salines; but when, after having duly administered these remedies, symptoms of cerebral disorder continue, the iodide should then be employed. The writer, in concluding, is satisfied that the iodide of potassium never produces any bad effects, though it may fail to do good.—(*Edinburgh Med. Jour.*, Dec. 1859.)

